# Leptomeningeal metastasis in a marginal zone lymphoma, presenting as a delirium: case report

**DOI:** 10.1186/s12877-020-01608-6

**Published:** 2020-06-17

**Authors:** Lisa Dreessen, Nicolas Maréchal, Michel Verheyden, Ann De Becker, Kristin Jochmans, Tim Vanderhasselt, Bert Bravenboer, Ingo Beyer

**Affiliations:** 1 Department of Geriatric Medicine, Vrije Universiteit Brussel (VUB), Universitair Ziekenhuis Brussel (UZ Brussel), Laarbeeklaan 101, 1090 Brussels, Belgium; 2 Department of Internal Medicine, Vrije Universiteit Brussel (VUB), Universitair Ziekenhuis Brussel (UZ Brussel), Laarbeeklaan 101, 1090 Brussels, Belgium; 3 Department of Hematology, Vrije Universiteit Brussel (VUB), Universitair Ziekenhuis Brussel (UZ Brussel), Laarbeeklaan 101, 1090 Brussels, Belgium; 4 Department of Radiology, Vrije Universiteit Brussel (VUB), Universitair Ziekenhuis Brussel (UZ Brussel), Laarbeeklaan 101, 1090 Brussels, Belgium

**Keywords:** Case report, Delirium, Leptomeningeal metastasis, Marginal zone lymphoma

## Abstract

**Background:**

Hematologic malignancies can spread to the central nervous system (CNS), either as focal lesions or as leptomeningeal disease. Marginal zone lymphoma (MZL) is a low-grade non-Hodgkin lymphoma and generally presents as an indolent disease. This case report illustrates an unexpected diagnosis of leptomeningeal metastasis in an MZL, presenting as a delirium without B symptoms, pronounced hematologic progression or abnormalities on cerebral imaging.

**Case presentation:**

An 80-year-old patient with a medical history of monoclonal B-cell lymphocytosis (MBL) with a clone indicative for an MZL, presented to the emergency and the geriatric departments with a recent cognitive deterioration and behavioral changes. MMSE score was 18/30. After excluding the most common etiologies through classical work-up including a normal head magnetic resonance imaging, a lumbar puncture was performed. In the cerebrospinal fluid an elevated protein level and increased lymphocyte count were identified, whereas beta-amyloid and tau protein levels were normal. Immunophenotyping of the lymphocytes confirmed CNS invasion by the MZL clone. Staging revealed mild splenomegaly. Prednisolone, intrathecal and systemic chemotherapy were initiated, leading to quick cognitive improvement with a final MMSE score of 28/30.

**Conclusions:**

To the best of our knowledge a delirium in an older patient due to leptomeningeal disease in MZL has never been described. To date, rare reports of CNS invasion by MZL describe focal intracranial lesions. After exclusion of common etiologies, physicians should remain vigilant when confronted with a patient with history of MBL presenting neurological symptoms. This case illustrates the importance of low threshold for lumbar punctures in this population, also for those patients with normal imaging studies.

## Background

Malignancies can cause central nervous system (CNS) involvement, either presenting as focal lesions or as leptomeningeal metastasis. The latter mechanism, also called neoplastic meningitis, is mostly seen as a late-stage complication of breast cancer, lung cancer or melanoma, and less frequently in hematologic neoplasms; mainly non-Hodgkin lymphoma, acute lymphoblastic leukemia or multiple myeloma. Leptomeningeal disease is usually caused by multifocal metastases to the leptomeninges [1, 2]. In 70–80% of the cases the diagnosis of leptomeningeal metastasis can be made using high quality T1-weighted magnetic resonance imaging (MRI). But cerebrospinal fluid (CSF) cytology for detection of malignant cells is the diagnostic gold standard [1].

A monoclonal B-cell lymphocytosis (MBL) is a rather indolent but frequently described condition that is most often diagnosed incidentally in the otherwise healthy older adult. The diagnosis is based on the identification of a clonal B-cell lymphocyte population persisting over a three-month period, without any lymphadenopathy or organomegaly, nor with an associated autoimmune or infectious disease [3, 4]. MBL is subclassified into three groups, the most common form being an MBL with a chronic lymphocytic leukemia (CLL)-like phenotype, for which it is assumed that it is a possible precursor for CLL. The other groups are an atypical CLL phenotype and a non-CLL phenotype [3]. Thus, MBL is a potentially pre-malignant condition for which regular follow-up should be provided [5, 6].

A clonal B lymphocytosis of marginal zone origin (CBL-MZ) describes specific MBL cases of non-CLL phenotype with features suggestive of a marginal zone origin [7], and can be considered as a potential pre-malignant condition that may or may not evolve into a marginal zone lymphoma (MZL) [5]. This report illustrates a diagnosis of leptomeningeal disease or, more specifically, a leptomeningeal lymphomatosis. It was caused by an MBL evolving to an MZL that presented as a persisting delirium without B symptoms or any abnormalities on radiologic examinations of the CNS.

## Case presentation

An 80-year-old, community-dwelling, Caucasian male patient presented at the emergency department with confusion, irritability, word finding difficulties, impaired concentration, disruption of circadian rhythm and a mild tremor. The symptoms had appeared suddenly 1 month earlier and were fluctuating but progressive up until presentation to the hospital.

The patient’s medical history included arterial hypertension, ischemic cardiomyopathy, chronic kidney disease stage 3a, type 2 diabetes mellitus, chronic length-dependent sensorimotor polyneuropathy and MBL. This MBL consisted of an indolent clone of a non-Hodgkin phenotype, indicative for a marginal zone origin (80%) and a smaller CLL-like phenotype clone (7%). At time of diagnosis of MBL, almost 3 years before the actual admission, the patient had no B-symptoms, no nodal or extra-nodal involvement nor organomegaly, so a watchful waiting policy was adopted, according to the prevailing guidelines.

His chronic treatment consisted of low dose of aspirin, bisoprolol, simvastatin, lorazepam, lisinopril and metformin. No changes to the medication were made in the last months prior to his admission. There was no previous history of smoking or alcohol consumption. The patient had worked until the age of 71 years as a commercial agent. His wife denied pre-existing cognitive disturbances. The patient mentioned an increased stress level due to a family conflict. No other medical or socio-environmental problems were detected during the assessment.

At the time of admission to the geriatric medicine ward, the general physical examination revealed no abnormalities, but the neurological examination showed word finding difficulties, a mild action tremor of the upper limbs, more marked on the left side, and a subtle postural instability. No signs of meningeal irritation were present.

Cognitive assessment revealed a Mini Mental State Examination (MMSE) score of 18/30, with decreased scores on orientation to time (2/5), orientation to place (4/5), memory recall (1/3), attention and calculation (3/5) and complex commands (3/5), where language and repetition testing (4/4) as well as immediate memory registration (3/3) were correct. He had a total score of 63/105 in the Cambridge Cognitive Examination (CAMCOG) neuropsychological battery, with subscale scores of 12/27 on memory, 24/30 on language, 5/12 on praxis, 5/7 on concentration, 4/8 on abstract thinking, 6/9 on perception, 1/2 on calculation and 6/10 on orientation testing.

Laboratory testing showed an increased total leucocyte cell count of 20.100/mm^3^ (reference range 3.600–9.600/mm^3^), with 13.507/mm^3^ (reference range 1.200–3.500/mm^3^) or 67.2% (reference range 19–44%) lymphocytes, compared to 17.000/mm^3^ or 59.5% leucocytes 1 year before. His hemoglobin level was normal at 13.7 g/dL, as well as the platelet count of 202.000/mm^3^. Kidney function was stable with a urea/creatinine ratio of 43/1.29. There were no signs of infectious or autoimmune disease, no liver dysfunction or electrolyte disorder.

**Fig. 1:**
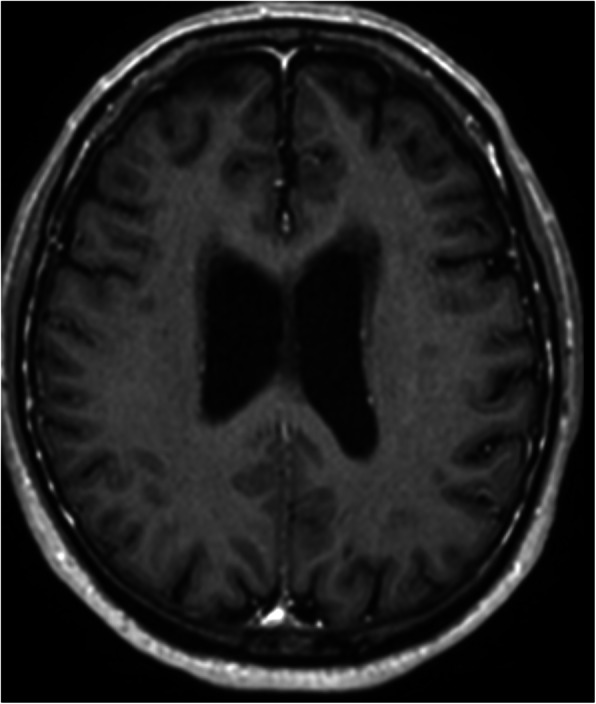
MRI image 3D T1 TFE + gadolinium demonstrating some periventricular white matter lesions and a mild cortical and subcortical atrophy. There is no abnormal meningeal enhancement

Electroencephalogram (EEG), 24-h Holter monitoring and carotid duplex ultrasound were considered normal. Computed tomography (CT) of the brain did not reveal any acute pathology. Cerebral MRI showed periventricular and subcortical white matter lesions and a moderate corticosubcortical and cerebellar atrophy, but no recent lesions (Fig. 1).

A non-traumatic lumbar puncture (LP) was performed; analysis of the CSF showed an elevated protein count of 1.010 mg/L (reference range 260–790 mg/L) and a leucocyte count of 144/mm^3^ (reference range 0–5/mm^3^) with 98% morphologically normal lymphocytes (reference range 40–80%). Glucose level was slightly lowered with a CSF-to-serum glucose ratio of 57% (normal CSF-to-serum glucose ratio of > 60%). Lactate level was at the cut-off of normality (1.7 mmol/L with reference value < 1.7 mmol/L). A normal cerebrospinal fluid biomarker profile (T-tau, Aβ_1–42_, P-tau_181P_ and 14–3-3Y) was identified, as such not suggestive for Alzheimer’s disease or Creutzfeldt-Jakob’s disease. An auto-immune and infectious work-up, including syphilis serology and an extensive set of microbiological cultures, was negative.

**Fig. 2:**
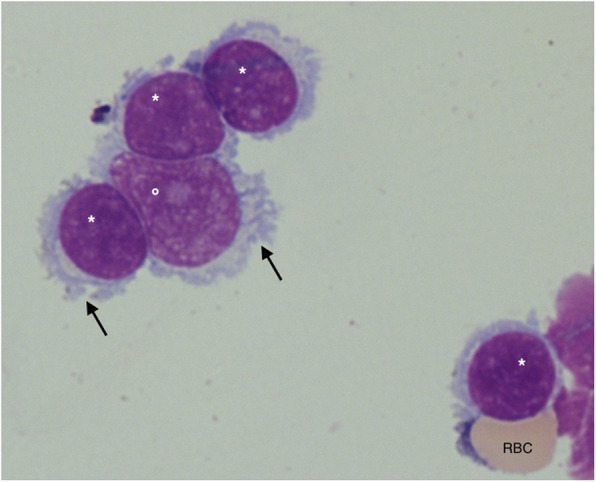
CSF microscopy showing one medium- (°) and four small-sized (*) lymphoid cells with clumped and sometimes irregular nuclear chromatin. They have a moderately abundant, slightly basophilic cytoplasm with fine protrusions (arrows). The small lymphoid cell in the lower right corner has an adjacent red blood cell (RBC)

A flow cytometry on CSF identified a voluminous monoclonal B-cell population with characteristics of the known marginal zone clone, confirming CNS invasion of this non-Hodgkin’s lymphoma (Fig. 2). Staging by chest and abdominal CT showed mild splenomegaly, not described on a CT 3 years earlier. Thus, the primary diagnosis of MZL with leptomeningeal lymphomatosis and splenic invasion was established.

During the in-hospital observation phase of 2 weeks at the geriatric department, the patient showed a fluctuating cognitive status and hyperactive behavior. Main disturbances were a distractibility, nocturnal agitation and insomnia, orientation and memory loss, emotional dysregulation with episodes of abundant crying, frontal behavior with unpredictable verbal aggression toward his wife and the caregivers, slight delusions of intentional harm, depressive thoughts and a remarkable variability in cooperation. Confusion Assessment Method (CAM) and Diagnostic and Statistical Manual (DSM)-V criteria for delirium were present, but the symptoms did not require an antipsychotic treatment. A proactive, non-pharmacological management of delirium was applied. This included, amongst others, early mobilization, avoidance of catheters or physical restraints, providence of orientation cues, behavioral therapy and frequent family visits. A therapeutic trial with mirtazapine was started in addition to psychological support for a mild depression. An attempt to reduce the dose of his chronic treatment with lorazepam was not successful. Despite these pharmacological and non-pharmacological measures, symptoms remained.

As diagnosis of stage IV MZL was made, cytoreductive therapy was initiated. The patient was treated with a short course of oral dexamethasone and four cycles of intrathecal chemotherapy consisting of methotrexate 15 mg plus six cycles of systemic chemotherapy R-COP (rituximab - cyclophosphamide - vincristine - prednisone). The patient’s general condition and age were considered as contraindications for systemic methotrexate therapy, splenectomy and autologous stem cell transplantation.

After 6 days of oral treatment with dexamethasone a subtle but clearly progressive improvement of the cognitive and behavioral status was seen, with normalization of the sleeping pattern, adequate orientation and a stabilization of the emotional regulation. Fourteen days after initiation of dexamethasone and 7 days after the first intrathecal chemotherapy, MMSE score had improved to 28/30 (memory recall score remaining 1/3). An important decrease of the CSF leucocyte count to less than 2/mm^3^ was documented two and a half months after initiation of therapy, suggestive for a good response on intrathecal chemotherapy. The therapy was well tolerated.

The patient’s mild tremor did not change significantly during hospitalization or afterwards, indicating that he probably suffered from an essential tremor. No follow-up on neurology was desired by the patient, as these symptoms were not experienced as disabling.

Currently, more than 2 years after initiating therapy, no clinical or biological signs of disease progression have been identified. Neither the patient, nor his family report a cognitive deterioration.

## Discussion and conclusions

Non-CLL-like monoclonal B cell lymphocytosis rarely progresses towards indolent non-Hodgkin lymphoma like marginal zone lymphoma [3, 8]. In general, marginal zone lymphoma is also indolent [5, 8, 9] and rarely spreads to the CNS [10]. To date, reports of CNS invasion by marginal zone lymphomas describe focal intracranial lesions, for instance mucosa-associated lymphoid tissue (MALT) subtypes mimicking meningioma [11–13] and dural MZL [14].

In this case report, the main presentation was a delirium that appeared suddenly, persisted over several weeks and resolved when the underlying medical condition was treated. Screening for delirium as part of the Comprehensive Geriatric Assessment (CGA) was positive, based on the CAM criteria [15] of an acute onset and fluctuating course, inattention and disorganized thinking. According to DSM-V [16, 17], a delirium is described as a disturbance in attention and awareness. It develops over a couple of days and is not explained by a pre-existing neurocognitive disorder. It tends to fluctuate and is accompanied by an additional disturbance in cognitive functioning. This neurobehavioral syndrome is a direct physiological consequence of another medical disorder. In this case a CNS involvement by a non-Hodgkin lymphoma was identified as the underlying condition. Soon after initiating therapy, a subjective improvement in cognitive state and behavior, and an objective improvement in MMSE-score were seen, supporting the etiological relationship between the delirium and the hematological diagnosis.

To the best of our knowledge, a delirium in an older patient due to leptomeningeal metastasis by a marginal zone lymphoma has never been described.

Studies revealed a large range of predisposing and precipitating factors for delirium, as well as multiple mechanisms for the pathophysiology of delirium. Local and systemic inflammation on one hand, and direct brain damage due to neoplastic invasion of the CNS on the other, seem to be core precipitant factors to the development of delirium in this case. Leptomeningeal metastasis of a lymphoma can present with a variety in neurological signs and symptoms since lymphoma cells in the subarachnoid space gain access to all portions of the CNS and cause direct brain damage [18–20]. Elevations of immunoglobulins and cytokines such as interleukin IL-6 in CSF, indicate a local, intrathecal immune activation in patients suffering leptomeningeal disease [18, 21]. Furthermore, a systemic inflammatory response can be expected due to the underlying hematological malignancy. Studies have shown that peripheral inflammation, i.e. increase and decrease of certain cytokines, influence brain functioning, directly through neurodegeneration, and indirectly by altering sleep, nutritional intake and affecting neurotransmission [18, 22, 23].

These acute precipitant factors are each having their effects depending on a patient’s specific fragility to the development of delirium [23, 24]. In this case report, amongst others, possible predisposing factors were his increased age and age-related factors of CNS decline, such as periventricular white matter lesions, and a depression. According to the systems integration failure hypothesis [24], this combination of precipitant factors and specific substrates lead to a complex web of pathways of neurotransmitter dysfunction and disruption in neuronal network connectivity, resulting in acute brain failure, presented as a delirium.

Several limitations of this case report can be identified. There is a lack of comparison to reference neuropsychological test results before the onset of the delirium and after completing hematological treatment. Pre-existing dementia is a risk factor for delirium, and on the other hand, delirium is to be considered as an independent risk factor for long-term cognitive decline and dementia [23]. The presumption of a persisting good cognitive functioning is only based upon subjective findings by the hematologists, who are still following the patient on an ambulatory basis, and the family. As memory recall scores remained low despite recuperation on other MMSE test levels after 2 weeks of oncological treatment, the lack of cognitive follow-up is particularly regretful. Furthermore, it is uncertain what role other predisposing and precipitating factors played in the onset of the symptoms, amongst others the mild depression. However, the significant increase of the MMSE score related to the oncological treatment, the significant decrease in caregiver burden and the subjective increase in quality of life in this patient are all arguments for adequate diagnosis and treatment.

After exclusion of common etiologies, physicians should remain vigilant when confronted with elderly with a prior history of monoclonal B cell lymphocytosis presenting neurologic signs as a confusional state. This case demonstrates that the threshold for a lumbar puncture for CSF cytology, in patients with a history of (pre) neoplastic diseases, should be low also for those with normal MRI studies and in absence of overt progression indicated by peripheral blood cell counts.

## Data Availability

Data sharing is not applicable to this article as no datasets were generated or analyzed during the current study.

## Abbreviations

CNS: Central nervous system
MZL: Marginal zone lymphoma
MBL: Monoclonal B-cell lymphocytosis
MMSE: Mini Mental State Examination
MRI: Magnetic resonance imaging
CSF: Cerebrospinal fluid
CLL: Chronic lymphocytic leukemia
CBL-MZ: Clonal B lymphocytosis of marginal zone origin
CAMCOG: Cambridge Cognitive Examination
EEG: Electroencephalogram
CT: Computed tomography
LP: Lumbar puncture
CAM: Confusion Assessment Method
DSM: Diagnostic and Statistical Manual
R-COP: Rituximab - cyclophosphamide - vincristine – prednisone
MALT: Mucosa-associated lymphoid tissue
CGA: Comprehensive Geriatric Assessment
